# Sleepless in America: A social sensing study of pandemic-era sleeplessness using nighttime social media data

**DOI:** 10.1371/journal.pone.0356547

**Published:** 2026-09-09

**Authors:** Xi Gong, Lin Liu, Yujian Lu, Guiming Zhang, Xiao Huang, Yan Lin

**Affiliations:** 1 Department of Biobehavioral Health, The Pennsylvania State University, University Park, Pennsylvania, United States of America; 2 Institute for Computational and Data Sciences (ICDS), The Pennsylvania State University, University Park, Pennsylvania, United States of America; 3 Department of Computer Science, University of New Mexico, Albuquerque, New Mexico, United States of America; 4 Department of Geography & Environmental Studies, University of New Mexico, Albuquerque, New Mexico, United States of America; 5 UNM Center for the Advancement of Spatial Informatics Research and Education (ASPIRE), University of New Mexico, Albuquerque, New Mexico, United States of America; 6 Department of Geography & the Environment, University of Denver, Denver, Colorado, United States of America; 7 Department of Environmental Sciences, Emory University, Atlanta, Georgia, United States of America; 8 Department of Geography, The Pennsylvania State University, University Park, Pennsylvania, United States of America; 9 Social Science Research Institute (SSRI), The Pennsylvania State University, University Park, Pennsylvania, United States of America; Queen Mary University of London, UNITED KINGDOM OF GREAT BRITAIN AND NORTHERN IRELAND

## Abstract

Sleeplessness is a widespread public health concern that was further intensified during the COVID-19 pandemic, yet large-scale, real-time monitoring of sleep disturbance remains limited. This study introduces a scalable social sensing framework that leverages temporally filtered nighttime social media activity (10:00 p.m. - 6:00 a.m. local time) to infer population-level patterns of sleep disturbance. To demonstrate the framework, we conducted a comprehensive analysis of geotagged tweets posted by users in the United States from March 1, 2020 to June 30, 2021, using pre-pandemic tweets as a baseline for comparison. Spatial, temporal, sentiment, and topical patterns were analyzed to characterize nighttime sleep disturbances at the U.S. state level. Three distinct temporal patterns of nighttime disturbance were identified during the pandemic, including single peak, multiple peaks, and smooth patterns, indicating heterogeneous impacts across states. Correlations between nighttime tweet ratios and daily new COVID-19 case counts varied across states and pandemic stages. In addition, more negative emotions expressed in nighttime tweets were associated with increased sleep disturbance. Nighttime tweets were more likely to focus on local news and events. Greater topical diversity among the population was associated with reduced negative emotional impacts during the pandemic. Overall, the findings demonstrate that nighttime social media data provide an effective and scalable social sensing approach for examining population-level patterns of potential sleep disturbance across large geographic regions and extended time periods. Beyond sleep-related applications, this framework offers a transferable method for monitoring other forms of nighttime human activity and broader social dynamics, including crime, disaster response, and social unrest.

## Introduction

It is estimated that approximately one-third of Americans have experienced sleeplessness, commonly referred to as insomnia or insufficient sleep [1,2]. Chronic sleeplessness is associated with substantial adverse health outcomes, including depression, obesity, and an elevated risk of injury [3,4], as well as broader societal impacts such as reduced productivity [5,6]. Research has shown that sleep duration and sleep patterns are influenced by a range of demographic and socioeconomic factors, such as gender, race/ethnicity, employment status, income, education, and marital status [7–10]. Additionally, societal events, such as pandemics, conflicts, protests, and natural disasters, can substantially exacerbate sleeplessness and disrupt typical sleep patterns [11–13].

Traditionally, researchers have relied on survey-based approaches to evaluate the level of sleeplessness, which can offer direct and reliable measurements [14,15]. However, the labor-intensive and time-consuming nature of surveys often results in limited sample sizes and restricted observation periods, which may limit their ability to capture population-level sleep disturbance patterns. Satellite-derived nighttime light imagery has emerged as an efficient tool for capturing spatiotemporal patterns of human nighttime activities over large areas and extended periods. Nevertheless, such images primarily reflect macro-level social and natural processes rather than micro-level human behaviors. Consequently, nighttime light images are most commonly used to monitor infrastructure development, detect natural fires, identify human settlements, and estimate socioeconomic conditions [16–18]. Because of this indirect relationship, nighttime light imagery provides limited insight into sleep patterns or sleep disturbance.

A more efficient and accurate approach is needed to collect large samples of sleep patterns, with social sensing using social media data emerging as a promising solution. Social sensing refers to the utilization of sensing and data collection methodologies to gather information directly from individuals or through devices acting on their behalf [19]. In recent decades, social sensing has become increasingly prominent in social research, driven in part by the rapid growth of social media platforms such as Facebook, Twitter, YouTube, and Instagram [20]. Social media platforms have large user populations, with each individual user effectively acting as a sensor [21]. The continuous generation of massive digital footprints on these platforms provides valuable data sources for observing and analyzing human activities and behaviors [20,22]. Compared with traditional surveys and satellite-derived nighttime light images, social media data offer larger sample sizes, providing a more comprehensive representation of the population.

This study introduces a novel framework that leverages nighttime social media big data to monitor and investigate population-level patterns of potential sleeplessness at a large scale. We define nighttime activity as those posted by typical individual users between 10 p.m. and 6 a.m. local time, while daytime activity constitutes those posted between 6 a.m. and 10 p.m. local time. The nighttime activity ratio is calculated as the percentage of posts during nighttime relative to the total number of posts sent within the same 24-hour period. In this study, we use the term “sleeplessness” operationally to refer to population-level nighttime wakefulness inferred from elevated nighttime tweeting activity. Although nighttime posting does not directly measure clinically defined sleep disturbance, insomnia, or poor sleep quality at the individual level, unusually high nighttime activity ratios may provide a population-level social sensing signal related to potential sleeplessness or shifts in typical sleep timing.

It is worth noting that research at the intersection of social media and sleep remains limited. Cellini et al. and Garett et al. studied the impact of social media usage around bedtime on sleep outcomes [23,24]. While Cellini et al. found that increased usage did not affect sleep habits [23], Garett et al. reported an association between social media usage and sleep quality among students [24]. Tandon et al. examined how social media usage driven by “fear of missing out” (FoMO) can negatively influence sleep quality [25]. Sakib et al. found that users’ social media interactions and content could help build predictive models for insomnia [26], but their analysis focused on social media posts throughout the entire day rather than specifically during nighttime hours. Collectively, these studies treat social media usage as a potential influencing factor on sleep patterns, rather than monitoring sleep disturbance directly using social media activities.

To demonstrate this framework, we utilize Twitter, now rebranded as X, as a special case. Twitter remains one of the most widely used social media platforms because of its ease of access, more structured messaging format, and high degree of interpersonal interaction relative to other platforms. As of October 2021, there are approximately 80 million Twitter users in the United States [27]. Each tweet (a message posted on Twitter) is accompanied by metadata, including user profile details, tweeting timestamp, and network connections with other users (directed followings, hashtags, at-mentions, and retweets) [28]. Additionally, a subset of tweets includes geotags, providing information on the tweet’s posting location [29]. These features constitute a valuable resource for observing and analyzing human social behavior in space and time. Furthermore, nighttime tweets generally exhibit lower levels of background noise than daytime tweets, as they contain fewer messages related to non-personal or automated content, such as commercial advertising, promotional posts, and live event broadcasts. As a result, Twitter data, and nighttime tweets in particular, offer an efficient means of collecting large samples of human behavior across extensive geographic regions for this study.

The complexity of sleeplessness was further intensified in 2020 with the onset of the global COVID-19 pandemic, which introduced heightened levels of uncertainty, anxiety, stress, and depression, along with concerns about personal safety and health for individuals and their loved ones. Moreover, the economic downturn stemming from the pandemic increased uncertainty related to employment, household finances, and social welfare systems [30]. Expanded media coverage of COVID-19 has also been associated with elevated levels of depression [31]. These emotional stressors may further disrupt regular sleep patterns [32,33]. During the pandemic, many U.S. counties implemented curfews and lockdown measures that substantially altered daily routines for numerous individuals [34]. Consequently, many people had to adapt to remote work and/or expanded caregiving responsibilities, often spending extended periods at home [35]. Such widespread disruptions likely exacerbated issues related to sleeplessness, particularly among vulnerable populations such as the older adults, children, ethnic minorities, individuals with psychiatric conditions, and those experiencing economic hardship [36]. In light of these intersecting factors, it is imperative to investigate sleeplessness within the context of the pandemic to elucidate its implications for public health and societal well-being. Therefore, the study applies nighttime social media data to examine sleep disturbance patterns in the United States during the COVID-19 pandemic.

This study introduces a novel framework that leverages nighttime social media big data to monitor and investigate sleep disturbance at a large scale, offering a promising direction for future research. Using sleep disturbance patterns in the United States during the COVID-19 pandemic as an illustration, the research innovatively integrates spatial, temporal, and sentiment perspectives to provide a comprehensive analytical approach. Specifically, it aims to address the following two questions: [1] How can nighttime social media data be systematically transformed into a reliable indicator of sleep disturbance? and [2] What do the resulting spatiotemporal patterns reveal about the resilience and vulnerability of populations during a global crisis? This study demonstrates the efficacy of nighttime social media big data, with tweets as a case study, in analyzing human sleep behavior. Furthermore, beyond sleep related applications, nighttime social media data may also be extended to detect and monitor a variety of other human activities and social dynamics, including but not limited to crime, disaster response, or social unrest.

### Data source and preprocessing

This study utilized historical Twitter data that were collected in real time via Twitter streaming application programming interfaces (APIs) and archived in the Internet Archive Database (IAD) [37]. The streaming API captures approximately 1% of all publicly available tweets. Although the first confirmed case of COVID-19 in the United States was reported on January 21, 2020 [38], the pandemic was officially declared in March 2020, marking the onset of widespread media coverage and heightened public attention [39]. Accordingly, Twitter data from March 1, 2020, to June 30, 2021, were analyzed to examine sleep patterns during the COVID-19 pandemic. To enable comparative analysis, Twitter data from the pre-pandemic period of 2017–2018 were also collected and combined to establish a comprehensive pre-pandemic baseline dataset. Tweets containing geotags (e.g., geographic coordinates, addresses, and points of interest [POIs]) within the United States were extracted from the original datasets. These geotags were subsequently geocoded using the Nominatim API from OpenStreetMap [40]. In total, 89.3% of geotagged tweets during the pandemic (3,460,035 tweets) and 87.6% of geotagged tweets from the pre-pandemic period (4,792,688 tweets) were successfully geocoded to locations within the United States.

Furthermore, not all collected tweets were posted by common individual users; a substantial proportion was generated by public organizations, government agencies, celebrity management teams, internet bots, and similar entities. Chu et al. proposed the follower–followee ratio (FFR) as a straightforward yet effective criterion for identifying common individual users, defining accounts with an FFR between 0.33 and 3 as highly indicative of such users [41]. In addition, common individual users rarely have more than 100,000 followers [41]. To restrict the analysis to common individual users, we excluded tweets from accounts with FFR values outside the 0.33 to 3 range or with more than 100,000 followers. Consequently, 2,477,809 geotagged tweets from the pandemic period (71.6% of all geotagged pandemic-period tweets) and 3,733,843 geotagged tweets from the pre-pandemic period (77.9% of all geotagged pre-pandemic-period tweets), posted by common individual users, were retained for analysis.

**Fig 1:**
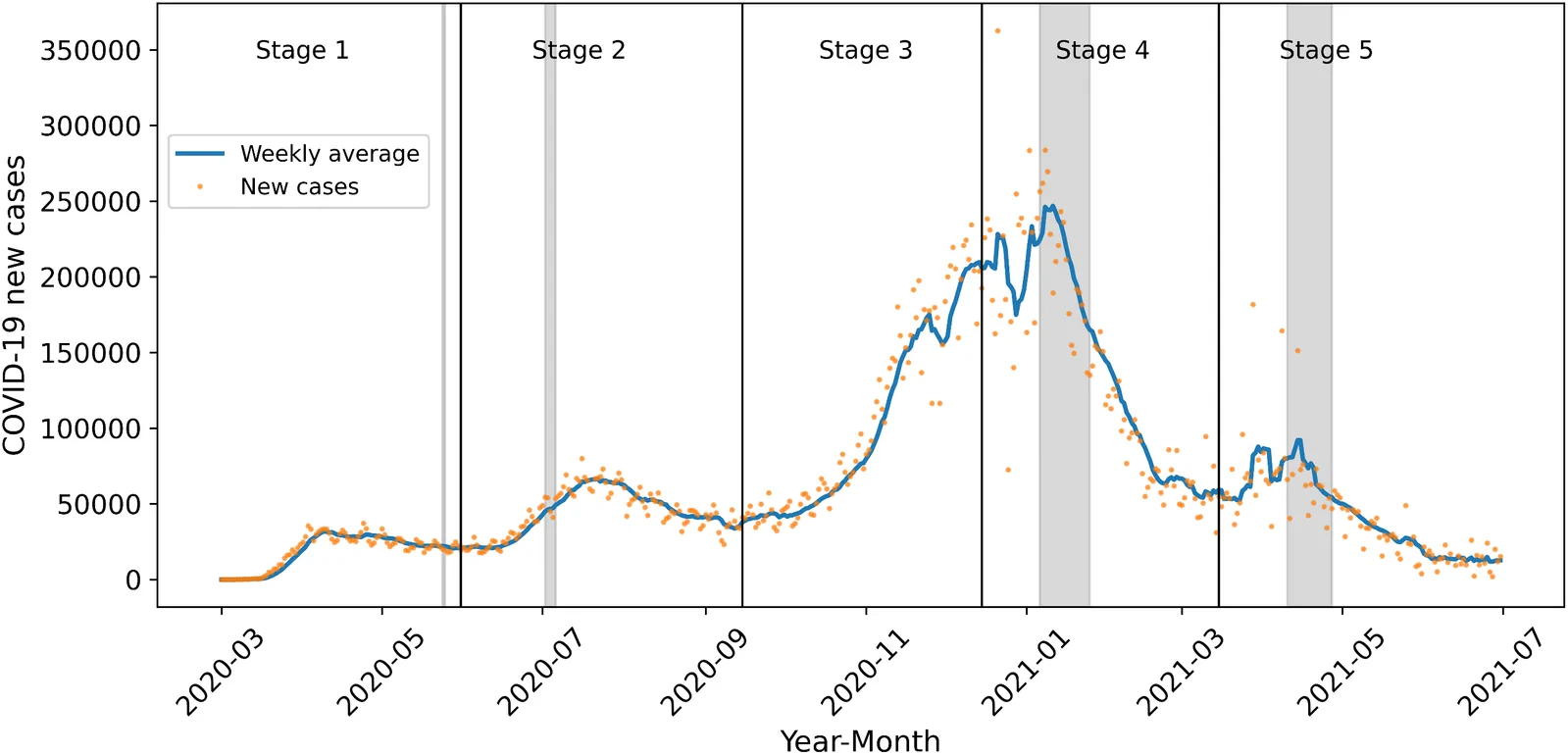
Trend in COVID-19 daily new cases in the United States from March 2020 to June 2021.

Several days are missing from the historical Twitter dataset collected during the pandemic, likely due to interruptions in the execution of the sampling code, such as power outages, exceeding API quotas, or other unforeseen circumstances. For non-consecutive missing days where data from both the preceding and following days are available, we estimate tweet statistics for the missing day (e.g., the nighttime tweet ratio) using the mean values of the two adjacent days. In contrast, consecutive missing days create intervals for which data are unavailable. Following this procedure, only four missing time intervals during the pandemic remain in this dataset: May 24–25, 2020; July 2–6, 2020; January 6–25, 2021; and April 10–27, 2021 (grey bands in Fig 1).

**Fig 2:**
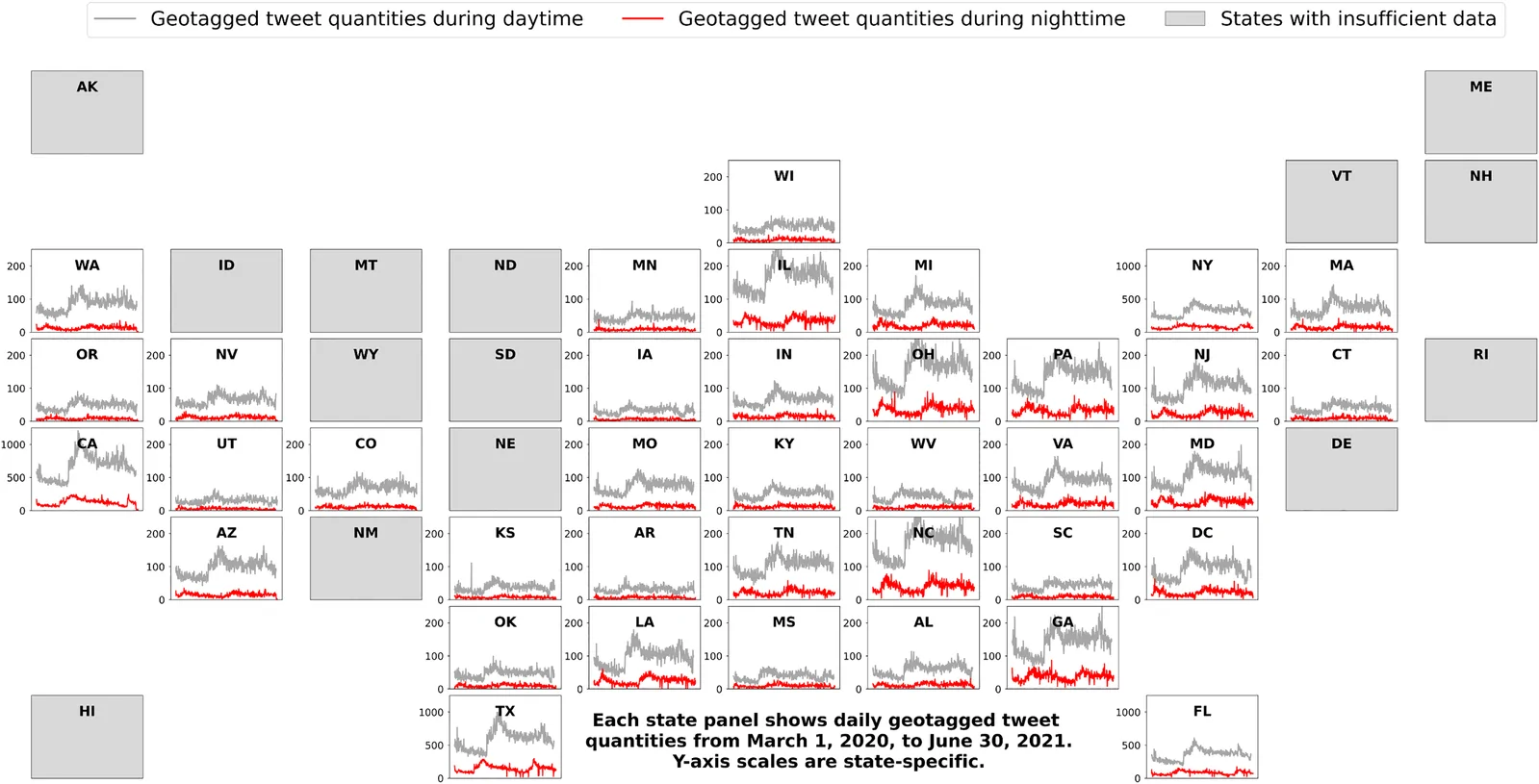
Daily geotagged tweet quantities during daytime and nighttime in U.S. states from March 2020 to June 2021.

As we aim to utilize daily nighttime tweet ratios at the state level as population-level indicators of sleep disturbance, it is essential to ensure a sufficient number of daily geotagged tweets for each state to achieve statistical reliability in calculating the ratio. To avoid unreliable estimates resulting from limited tweet counts, we applied minimum data requirements before calculating nighttime tweet ratios. Specifically, a state was included only if it contained more than 30 geotagged tweets per day. Given that nighttime tweets typically account for approximately 18–20% of daily tweets, this threshold corresponded to an expected five to six nighttime tweets and reduced the likelihood that nighttime ratios would be driven by only one or two observations. We also required each state to have more than 10 valid days per month. This threshold was selected to balance stability of monthly estimates and geographic coverage. Requiring a greater number of valid days would substantially reduce the number of states available for analysis; for example, increasing the requirement to at least 15 valid days per month reduced the number of eligible states to only 21. Conversely, allowing months with too few valid days could produce unstable and unreliable estimates of monthly nighttime activity patterns. Based on these criteria, 37 states with at least ten valid months between March 2020 and June 2021 were selected for further analysis, as shown in Fig 2. Other states were excluded because they had only 1–8 valid months during this period. The final dataset comprises 2,395,310 tweets during the pandemic (including 1,963,068 daytime tweets and 432,242 nighttime tweets) and 3,599,808 tweets from the pre-pandemic period (including 2,882,568 daytime tweets and 717,240 nighttime tweets) for further analysis.

To examine the relationship between pandemic status and sleep disturbance, we also collected daily new COVID-19 case counts for each U.S. state from March 1, 2020, to June 30, 2021, using data from the Centers for Disease Control and Prevention [42].

## Methods and results

This study first analyzed spatiotemporal patterns of sleeplessness in each state and identified similarities using clustering methods. Next, associations between daily new COVID-19 case counts and nighttime tweet ratios in each state were examined. Subsequently, temporal patterns of sentiment in tweet content across states were investigated using a machine learning approach. Finally, tweet content and hashtags were analyzed to explore potential factors shaping the observed patterns. Detailed methodologies and results are described in the following subsections.

### Spatial-temporal patterns of sleeplessness

**Fig 3:**
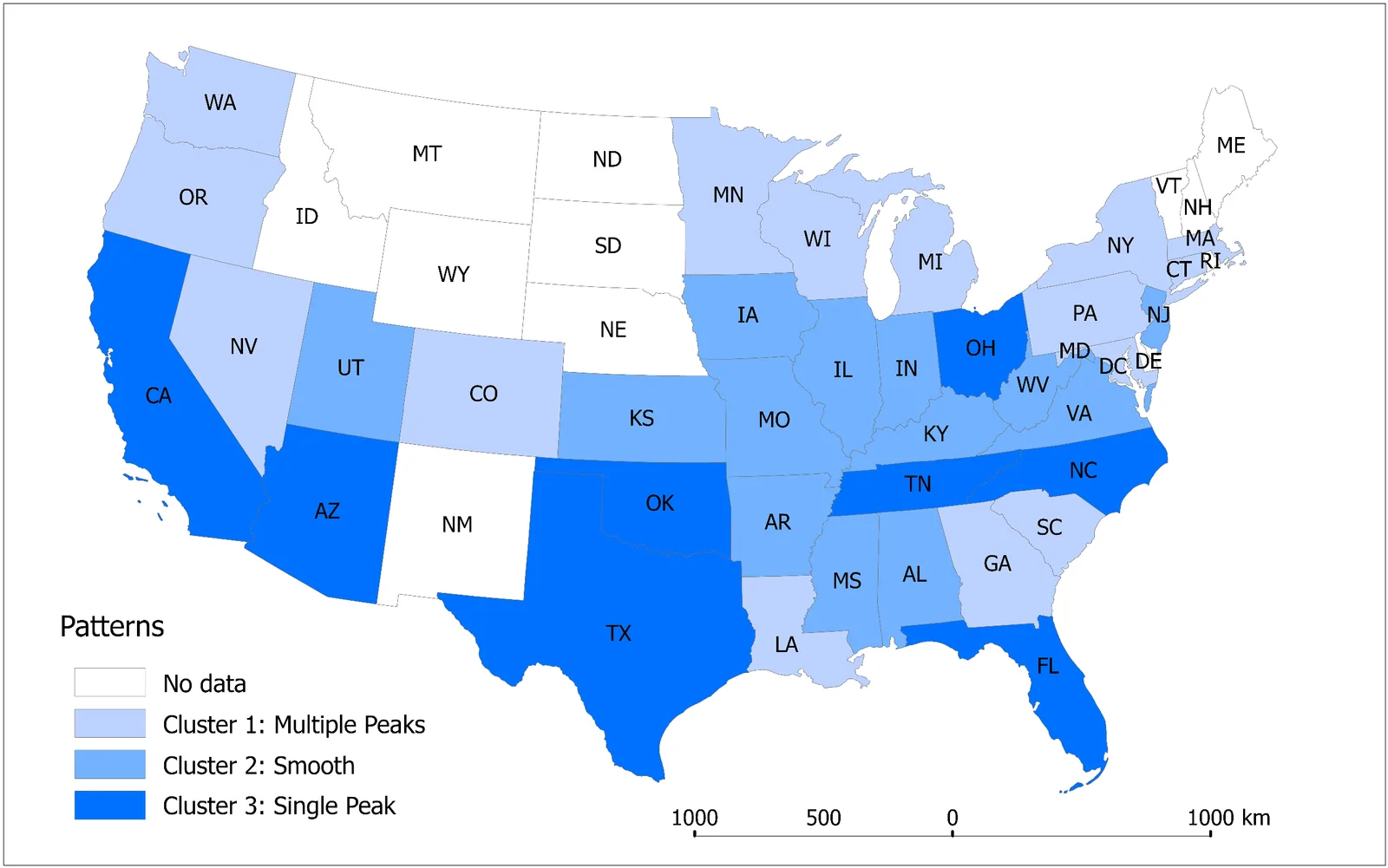
Spatial distribution of the patterns of monthly disturbance night percentages in U.S. states during March 2020 to June 2021.

**Fig 4:**
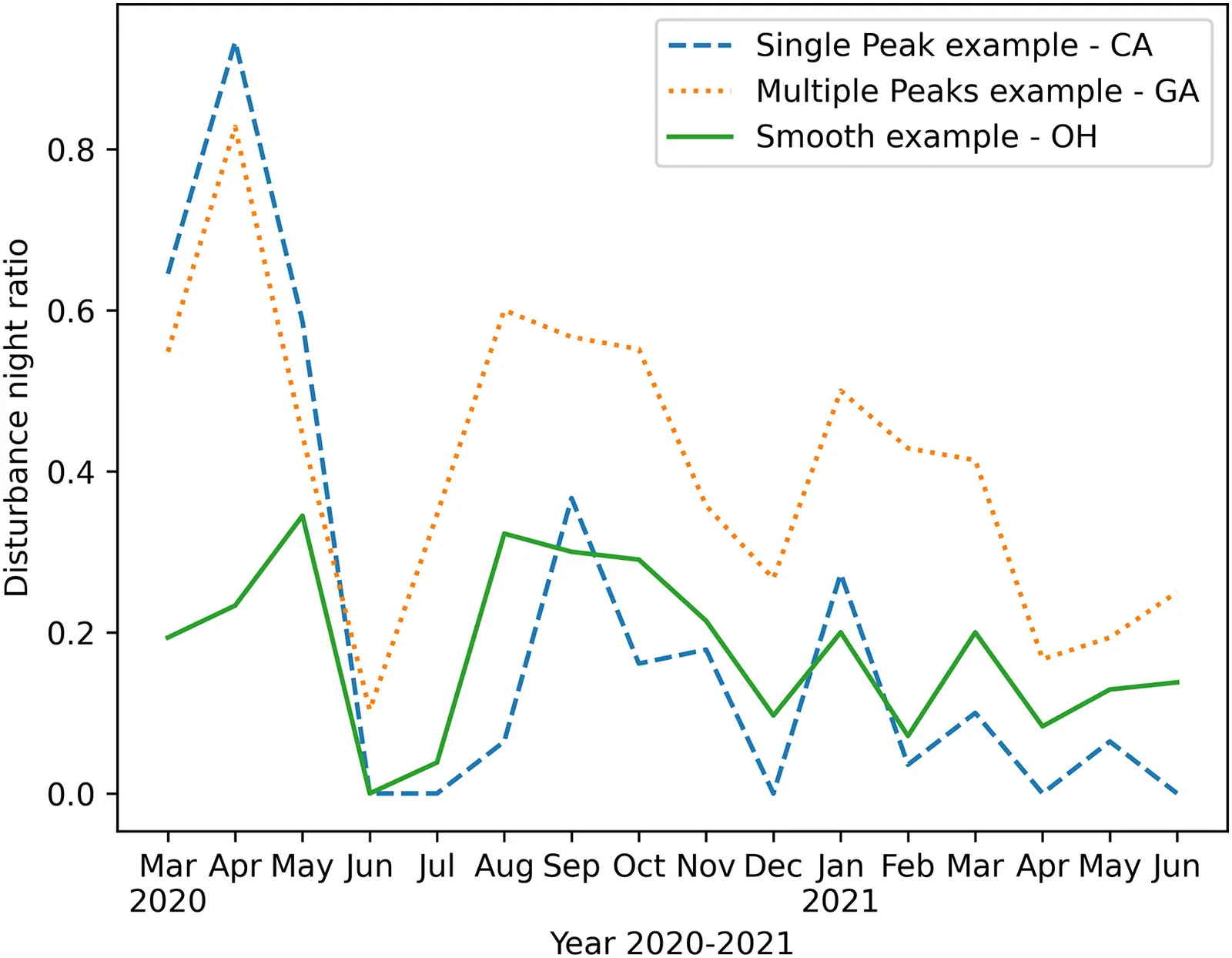
Pattern examples of monthly disturbance night percentages in U.S. states during March 2020 to June 2021.

We utilized monthly disturbance night patterns in each state to characterize spatial and temporal trends of sleeplessness in the United States. Disturbance nights were defined as days on which nighttime tweet ratios significantly exceeded their respective baselines. Pre-pandemic data from 2017 and 2018 were used to calculate, for each month, the mean daily nighttime tweet ratios from January to December for each state. Daily nighttime tweet ratios during the pandemic of each state were then compared with the corresponding pre-pandemic baseline values for the same state and calendar month. A pandemic-period night was classified as a disturbance night if its nighttime tweet ratio exceeded the corresponding pre-pandemic monthly mean, indicating increased nighttime tweeting activity relative to the historical pre-pandemic reference period. For each state, we calculated the percentage of disturbance nights in each month from March 2020 to June 2021, producing a 16-dimensional vector representing monthly sleeplessness trends. A higher percentage in a given month indicates that residents experienced more sleep disturbance during that month compared to others. Confidence intervals were not used in the disturbance-night classification; instead, reliability was controlled through the state-day and state-month coverage thresholds described above. Then, we applied the *K*-means clustering analysis [43] to the 16-dimensional vectors to identify the principal spatiotemporal patterns of sleeplessness across states (Fig 3 and Fig 4).

To determine the number of clusters, we evaluated values of *K* from 2 to 6 using the average silhouette score, treating each of the 37 states as one observation. The scores for *K* = 2–6 were 0.204, 0.145, 0.137, 0.122, and 0.105, respectively, indicating that *K* = 2 provided the strongest cluster separation according to this criterion. However, examination of the *K* = 2 solution showed that it merged states with visibly different temporal profiles into overly broad groups. In contrast, *K* = 3 separated the states into three distinct and interpretable temporal patterns: “Single Peak,” “Multiple Peaks,” and “Smooth.” When *K* = 4, the additional cluster primarily subdivided the multiple-peak pattern into double-peak and three-peak subgroups without substantially changing the overall interpretation. We therefore selected *K* = 3 as an interpretability-driven compromise between quantitative cluster separation and meaningful characterization of the temporal patterns (Fig 3). The “Single Peak” pattern is defined by a marked peak in disturbance night percentages during the initial months, followed by a decline to below 30% in subsequent months. California exemplifies this pattern, with disturbance night percentages exceeding 70% from March to May 2020 and subsequently decreasing to below 25% (Fig 4). This pattern indicates a pronounced alteration in sleep timing and duration during the early stages of the pandemic, likely driven by elevated anxiety and stress. As public awareness of the pandemic increased and recovery rates improved, individuals in these states appeared to return to more typical sleep patterns. In the “Multiple Peaks” pattern, disturbance night percentages fluctuate notably from month to month, characterized by multiple local maxima on the curve. Georgia exemplifies this pattern, with disturbance night percentages reaching 80% in April 2020, 65% in September 2020, and 70% in January 2021, respectively (Fig 4). This pattern suggests that sleep disturbance may have recurred across multiple periods during the pandemic. Because these peaks do not consistently align with COVID-19 waves, they may reflect the influence of multiple concurrent social, political, environmental, or local events in addition to the pandemic. For example, Minnesota experienced a substantial increase in disturbance night percentages during the George Floyd protests in May 2020, despite no unusually high daily new COVID-19 cases. The average nighttime tweet ratio in late May 2020 in Minnesota was significantly higher than the 2017 baseline (24.7% vs. 16.3%, *p* < 0.01), consistent with increased human mobility during this period [44]. Similarly, Wisconsin exhibited peak disturbance nights during the October 2020 election season, independent of any surge in COVID-19 cases. However, this interpretation should be viewed as a potential explanation rather than direct evidence of event-specific effects. Further event-level analysis is needed to determine which events contributed to these recurring peaks in future studies. States exhibiting the “Smooth” pattern maintained relatively low disturbance night percentages across all 16 months. Ohio exemplifies this pattern, with percentages consistently below 35% throughout the pandemic (Fig 4). This pattern indicates that the timing and duration of sleep in these states remained largely stable during this period.

### Nighttime tweet ratio and COVID-19 pandemic status

To examine whether nighttime tweeting activity was temporally associated with COVID-19 case trends, we analyzed the relationship between daily new COVID-19 cases and daily nighttime tweet ratios across U.S. states [38]. The pandemic study period (March 1, 2020–June 30, 2021) was divided into five stages based on national trends in daily COVID-19 cases (Fig 1). Stage 1 (March 1–May 31, 2020) represented the first wave; Stage 2 (June 1–September 15, 2020) the second wave; Stage 3 (September 16–December 15, 2020) the third wave; Stage 4 (December 16, 2020–March 15, 2021) the post-third-wave recovery period; and Stage 5 (March 16–June 30, 2021) the later study period, during which the Delta variant began to emerge.

For each state and each pandemic stage, we applied time-lagged cross-correlation [45] to examine whether fluctuations in COVID-19 cases were associated with changes in nighttime tweeting activity. Daily new COVID-19 cases were smoothed using a 7-day running average, and daily nighttime tweet ratios were calculated as described above. To account for possible delays between changes in COVID-19 case trends and changes in nighttime social media activity, we considered time lags of up to seven days. For each state-stage pair, we calculated the lagged correlation coefficients, associated *P*-values, and corresponding time lags.

To summarize these associations across states, we used a rule-based positive-association classification. For each state-stage pair, a correlation coefficient was retained only if its associated *P*-value was less than 0.05 and the selected correlation was positive. Non-significant correlations were set to zero. Significant negative correlations were also set to zero because the purpose of this analysis was specifically to identify periods in which nighttime tweet ratios increased in parallel with COVID-19 case trends. Therefore, the resulting patterns should be interpreted as descriptive patterns of positive association, rather than as a complete characterization of all possible relationships between COVID-19 cases and nighttime tweeting activity.

This approach produced a five-stage positive-association vector for each state. A notable feature of the results was that no state showed a significant positive selected correlation during Stage 3. Therefore, Stage 3 did not contribute to distinguishing positive-association patterns across states. We therefore summarized the temporal information by grouping Stages 1 and 2 as the early pandemic period and Stages 4 and 5 as the late pandemic period. For each state, early- and late-period indicators were defined based on whether at least one significant positive selected correlation was observed within the corresponding stages.

Based on the presence or absence of significant positive associations in the early and late periods, states were classified into four mutually exclusive positive-association patterns: Early-only positive, Late-only positive, Early–late positive, and No positive association. Early-only positive states had significant positive correlations only during Stages 1 and/or 2; Late-only positive states had significant positive correlations only during Stages 4 and/or 5; Early–late positive states had significant positive correlations in both periods; and No positive association states had no significant positive selected correlations across all five stages.

**Fig 5:**
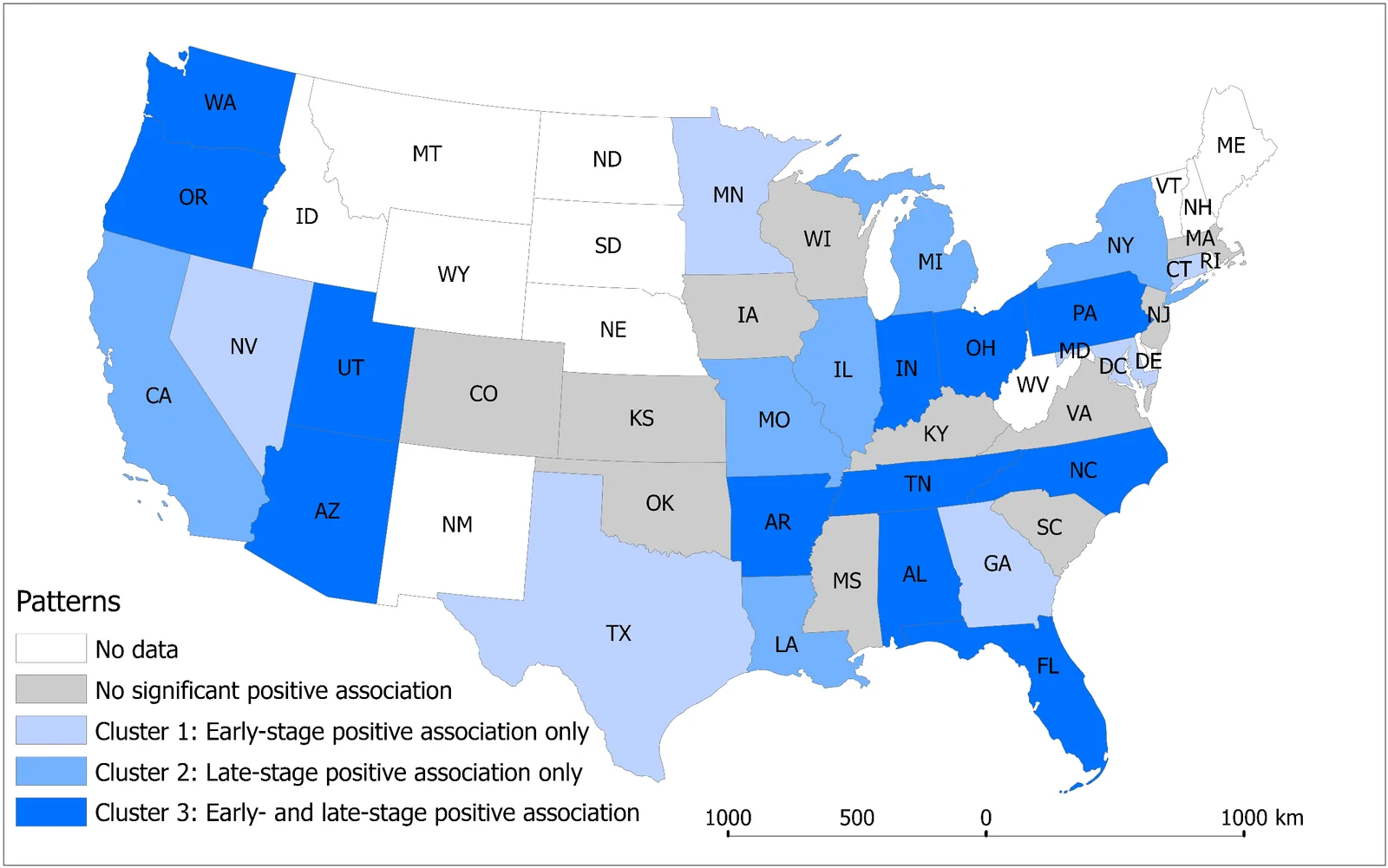
Patterns of associations between COVID-19 daily new cases and nighttime tweet ratios across U.S. states, March 2020 to June 2021.

All four positive-association patterns were observed, as shown in Fig 5. The Early-only positive pattern included seven states where nighttime tweet ratios were positively associated with COVID-19 case trends only during the early pandemic period. The average early-period correlation coefficient for this group was 0.292, while no significant positive correlation was observed in the late period. In these states, nighttime tweet ratios showed stronger alignment with COVID-19 case fluctuations during the early pandemic stages, but this association was not detected later in the study period. States in this group included Connecticut, Washington D.C., Georgia, Maryland, Minnesota, Nevada, and Texas.

The Late-only positive pattern included twelve states where significant positive correlations appeared only during the late pandemic period. This group had no significant positive correlation in the early period, while its average late-period correlation coefficient was 0.346. In these states, nighttime tweet ratios showed stronger alignment with COVID-19 case fluctuations during the later pandemic stages. States in this group included Alabama, Arkansas, Arizona, Florida, Indiana, North Carolina, Ohio, Oregon, Pennsylvania, Tennessee, Utah, and Washington. This pattern was observed across multiple regions rather than being confined to a single geographic area.

The Early–late positive pattern included six states with significant positive correlations during both the early and late pandemic periods. The average early-period and late-period correlation coefficients for this group were 0.351 and 0.353, respectively. In these states, nighttime tweet ratios showed positive alignment with COVID-19 case fluctuations across multiple pandemic phases, rather than only within one period. States in this group included California, Illinois, Louisiana, Michigan, Missouri, and New York.

The No positive association pattern included twelve states with no significant positive selected correlation under the selected lag structure and significance threshold. States in this group included Colorado, Iowa, Kansas, Kentucky, Massachusetts, Mississippi, New Jersey, Oklahoma, South Carolina, Virginia, Wisconsin, and West Virginia. For these states, nighttime tweet ratios did not show statistically detectable positive alignment with COVID-19 case fluctuations under the selected lag structure and significance threshold. This should not be interpreted as evidence that no relationship existed. Because statistical significance depends on sample size, variability, and the assumed correlation structure, some non-significant results may reflect limited statistical power, noisy state-level data, or relationships not captured by the selected lagged-correlation framework.

Overall, these results suggest that positive associations between COVID-19 case trends and nighttime tweeting activity were not uniform across states or pandemic stages. Instead, the associations appeared in different periods across different groups of states. These patterns provide descriptive evidence of spatial and temporal heterogeneity in pandemic-era nighttime tweeting activity, but they should not be interpreted as causal evidence that COVID-19 case trends directly caused sleep disturbance or nighttime social media activity.

This study identified three patterns of monthly disturbance nights (“Single Peak”, “Multiple Peaks” and “Smooth” as shown in Fig 3) and four patterns of correlations between nighttime tweet ratios and daily COVID-19 cases (Early-only positive, Late-only positive, Early–late positive, No positive association as shown in Fig 5). To assess whether these two sets of patterns were related, a chi-square test of independence was conducted (Table 1). With a *p*-value of 0.063 on chi-square test of independence, we failed to reject the null hypothesis of independence on 0.05 significance level, indicating that monthly disturbance night patterns and nighttime tweet ratio-pandemic status correlation patterns are statistically independent. One potential explanation is that the correlation patterns consider only daily COVID-19 case counts as a contributing factor to sleep pattern disruption. These patterns suggest that the pandemic, as a long-term event, affected sleep patterns primarily during specific stages of the study period, particularly at the onset of the first wave and during the Delta-variant wave. However, hashtag analysis indicates that COVID-19 was not the only dominant topic during the pandemic period in the United States. Monthly disturbance night patterns were also shaped by other concurrent trending events, such as presidential elections, sports events, and social protests, in addition to the pandemic. As a result, the two types of patterns capture different underlying influences, which explains their observed independence.

**Table 1:** Joint distribution of three monthly disturbance nights patterns and four correlation patterns between nighttime tweet ratios and daily COVID-19 cases in United States during March 2020 to June 2021.

		Correlation patterns between nighttime tweet ratios and daily COVID-19 cases				
		Early only positive †	Late only positive	Early-Late positive	No positive	Total
Monthly disturbance nights patterns	Single peak	1*	5	1	1	8
	Multiple peaks	6	3	3	4	16
	Smooth	0	4	2	7	13
	Total	7	12	6	12	37

* Cell value means the number of states.

† Early stage = stages 1–2; Late stage = stages 4–5. Stage 3 showed no significant positive associations. ‘Positive’ indicates a statistically significant positive association

### Sentiment of nighttime tweets

In analyzing the sentiment of the extensive tweet dataset, which includes posts in more than 50 languages, this study employed the BERT-base-multilingual-uncased-sentiment model developed by NLPTown and distributed via the Hugging Face platform [46]. The model is based on BERT (Bidirectional Encoder Representations from Transformers), a transformer-based deep learning architecture for contextualized language representation [47]. It is pre-trained for six languages: English, Dutch, German, French, Spanish, and Italian [46]. The model produces sentiment scores ranging from one to five stars for tweets in these languages, with lower values indicating more negative emotional content [46]. Because these six languages account for 86.7% of all tweets in both the pre-pandemic and pandemic periods, the sentiment analysis was restricted to this subset, and the remaining 13.3% of tweets were excluded from the analysis.

Comparing average sentiment scores in the United States between the pandemic and pre-pandemic periods for both nighttime and daytime tweets, we observed that both daytime and nighttime sentiment scores were lower during the pandemic than in the pre-pandemic period (Z-tests, *p* < 0.01). The average daytime sentiment score was 3.021 (SD = 1.823) during the pandemic and 3.088 (SD = 1.815) during the pre-pandemic period. For nighttime tweets, the average sentiment score was 2.935 (SD = 1.820) during the pandemic and 2.968 (SD = 1.818) during the pre-pandemic period. Notably, nighttime tweets consistently exhibited lower average sentiment scores than daytime tweets, both before and during the pandemic (Z-tests, *p* < 0.01), indicating a slightly more negative textual tone at night. Although these differences were statistically significant, their magnitudes were small relative to the variability in individual tweet sentiment scores. Therefore, the results indicate a modest shift in textual emotional tone rather than a large change in sentiment or direct evidence of anxiety or depressive symptoms.

To examine the relationship between tweet sentiment and nighttime tweeting behavior, two national-level daily time series were constructed for each period: the daily nighttime tweet ratio and the daily average sentiment score of nighttime tweets. During the pandemic period, Pearson correlation analysis showed a significant negative association between nighttime tweet ratio and nighttime sentiment (r = −0.190, *p* < 0.001), indicating that days with higher nighttime tweeting activity tended to have slightly lower average sentiment scores. In contrast, the corresponding pre-pandemic correlation was positive (r = 0.205, *p* < 0.001). This difference suggests that the relationship between nighttime tweeting activity and emotional tone changed during the pandemic period.

### Topics of nighttime tweets

We tracked the hashtags of nighttime tweets in the United States to identify major topics discussed during the pandemic period. The top 1,000 hashtags, ranked by frequency of occurrence, were manually classified into six thematic categories, each containing more than 100 unique hashtags. These categories include: [1] Entertainment, covering hashtags related to TV shows, movies, celebrities, and music (e.g., “BTS”, “Hollywood”, and “rap”); [2] U.S. presidential election, including hashtags such as “vote”, “debate”, “Biden”, “Trump”, and “MEGA”; [3] Pandemics, including hashtags such as “COVID”, “social distancing”, “COVID19”, and “quarantine”; [4] Sports, including hashtags like “NBA”, “NFL”, “UFC”, and “Superbowl”; [5] Protest, including hashtags like “GeorgeFloyd”, “ICantBreathe”, and “BlackLivesMatter”; and [6] Natural disasters, including hashtags like “earthquakes”, “wildfire”, and “storm”.

Within each trending topic category, the total number of hashtag occurrences in nighttime tweets posted in the United States from March 2020 to June 2021 was as follows: Entertainment (1,307 occurrences), U.S. presidential election (1,064), Pandemics (845), Sports (627), Protest (384), and Natural disasters (295). Pandemic-related hashtags ranked third among the six categories, indicating that the pandemic was not the sole dominant topic discussed on Twitter during the study period in the United States. Entertainment- and election-related hashtags were more prevalent nationwide during this timeframe. These findings suggest that, although the pandemic may have contributed to changes in nighttime tweeting activity and sleep-related disruption, the topics used in nighttime tweets were not necessarily centered on the pandemic itself.

**Fig 6:**
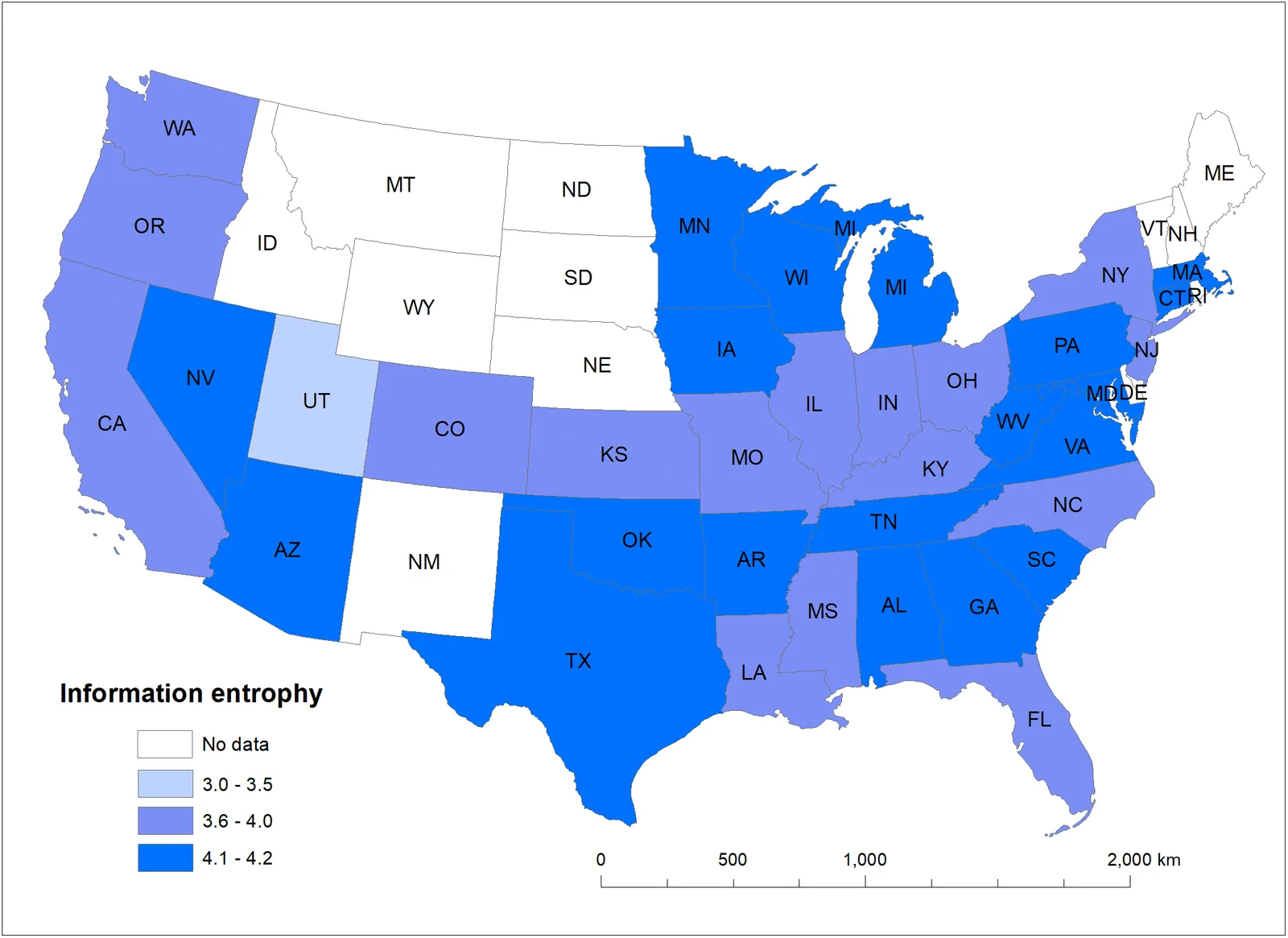
Topic-based information entropy of nighttime geotagged tweets posted in U.S. states from March 2020 to June 2021.

In each state, we identified the ten most frequently used nighttime hashtags during the study period and computed their information entropy to assess the diversity of public attention. The information entropy (*H*) is defined as Equation (1):


$$\begin{array}{c} H=-\sum_{i=\text{1}}^{n}p_{i}*logp_{i}\; \end{array}$$ (1)

where $p_{i}$ is the frequency of occurrence of the *i*-th hashtag among the top ten nighttime hashtags. Hashtags belonging to the same trending topic category were treated as identical. Lower information entropy values indicate a more concentrated focus on a limited set of topics, whereas higher values indicate a broader distribution of topics discussed on Twitter. Fig 6 presents the spatial distribution of topic-based information entropy across U.S. states during the pandemic. The Pearson correlation coefficient between information entropy and the state-level mean sentiment score is 0.58 (*p* < 0.01), indicating a statistically significant positive relationship. This finding suggests that states with more diverse nighttime topic engagement tend to exhibit higher average sentiment scores, implying that broader public attention across topics is associated with more positive emotional expression.

We further compared hashtags used in nighttime and daytime tweets on the same dates within each U.S. state. Our findings show that many trending events were discussed primarily at the local level during nighttime, but attracted broader, cross-state attention during the daytime. For example, a magnitude 4.6 earthquake struck California in September 2020. Daytime geotagged tweets referencing the earthquake were observed in 11 states, whereas nighttime geotagged tweets containing earthquake-related hashtags were found exclusively in California. Another example involves BTS, a popular South Korean boy band, who planned 2020 U.S. tour generated substantial online attention. Around the originally scheduled May 14 event in Orlando, Florida, BTS-related hashtags were widely distributed nationwide during the daytime, whereas 81.3% of the corresponding nighttime geotagged hashtags were posted in Florida. These examples indicate that nighttime tweets tend to focus more strongly on local news and events. As a result, sharp increases in nighttime tweet ratios within specific regions may signal local events that disrupt typical sleep patterns. Frequent mentions of specific topics in nighttime tweets from these areas may therefore serve as useful indicators for identifying and tracking such localized events.

## Discussion

This study uses nighttime tweets before and during the COVID-19 pandemic to examine spatial, temporal, emotional, and topical patterns of sleeplessness across the United States. The findings show that nighttime tweets can serve as a large-scale social sensing signal for examining population-level patterns of potential sleep disturbance across geographic areas and over time.

A comparison of nighttime geotagged tweets posted before and during the pandemic indicates a lower volume during the pandemic, with an average of 27,809.6 tweets per month, compared to the pre-pandemic period, with an average of 61,371.4 tweets per month (Fig S1). A plausible explanation is that, as the severity of the COVID-19 pandemic increased, many state governments implemented lockdown and curfew policies that restricted travel, social activities, and nightlife, which likely contributed to the decline in nighttime geotagged tweet postings. During the pandemic period, monthly nighttime geotagged tweet counts showed a general declining trend (Fig S1). In addition, a policy change implemented by Twitter in 2019 may have adversely affected the volume of geotagged tweets [48], potentially contributing to the overall decline observed during the pandemic period. Surprisingly, the nighttime tweet ratio was lower during the pandemic than before the pandemic, with mean ratios of 17.96% and 19.97%, respectively, and this difference was statistically significant in the paired-sample t-test (*p* < 0.001). Because this study identifies disturbance nights by flagging nighttime tweet ratios that exceed pre-pandemic baselines, the higher average nighttime tweet ratio in the pre-pandemic period raises the baseline. This makes it easier to identify nights with unusually elevated nighttime tweeting activity, which may indicate more intense disruption of typical sleep patterns.

Throughout the pandemic period, we examined multiple state-level factors potentially associated with negative emotions expressed in nighttime tweets, including educational attainment, median age, median household income, unemployment rate, the Gini index, and welfare expenditures, all obtained from the U.S. Census Bureau [49]. Among these variables, the state-level percentage of negative nighttime tweets showed a significant negative correlation only with total welfare expenditures (correlation coefficient = −0.61, *p* < 0.01). Welfare expenditures include the total expense on grants, food stamps, vouchers, Medicaid, and housing assistance within a specific state [50]. This negative correlation suggests that higher welfare spending may reflect stronger social support systems at the state level. As a result, residents in states with greater welfare support may feel more secure during the pandemic, which could mitigate negative emotional expression in nighttime tweets [51].

Because nighttime tweets during the pandemic covered a wide range of topics, changes in nighttime tweeting activity should not be attributed solely to COVID-19. Other concurrent social, political, environmental, and local events may also have contributed to the observed temporal patterns.

**Fig 7:**
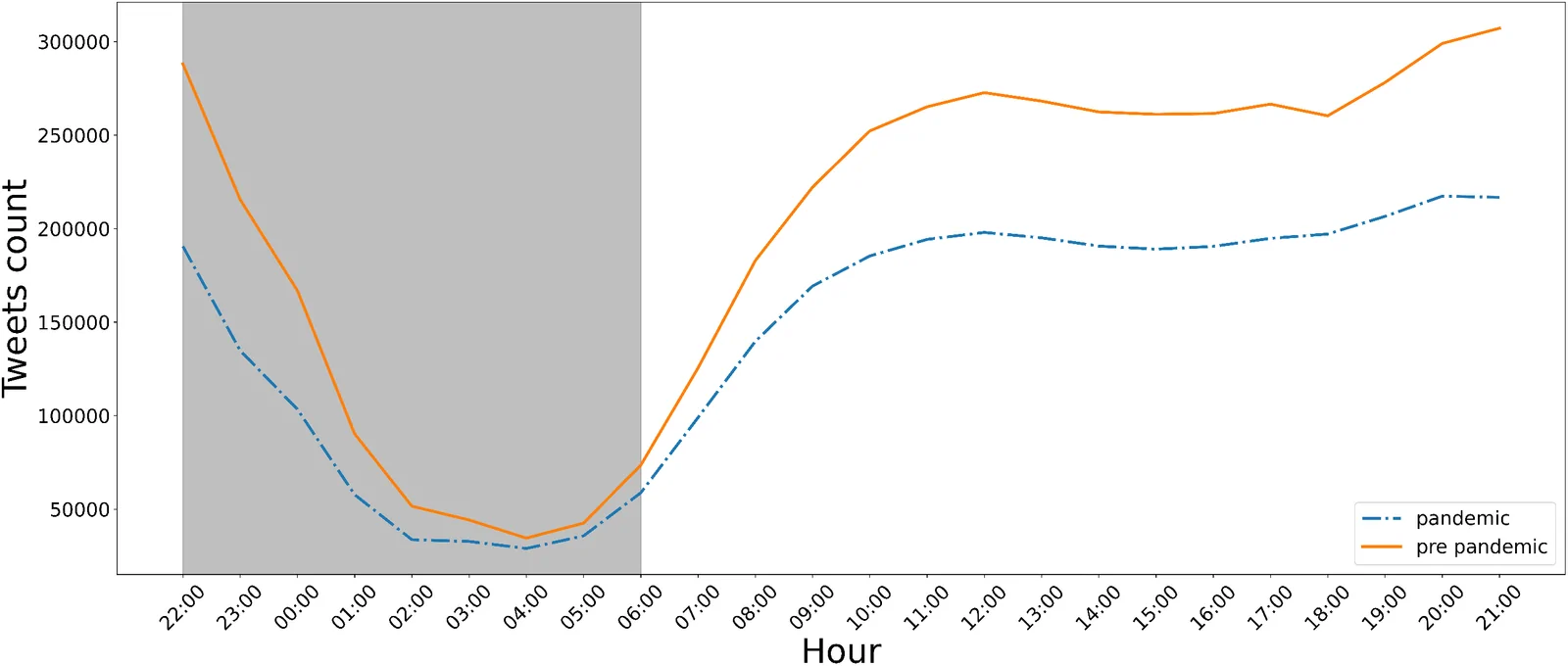
Geotagged tweets volumes by hour (local time) in United States before and during COVID-19 pandemic.

The Twitter Streaming API used in this study for data collection is limited to retrieving approximately 1 percent of all public tweets [52]. Although Twitter does not disclose its specific sampling strategy, prior research suggests that this low sampling rate can still capture key characteristics and temporal patterns of the full Twitter stream, supporting its use in sentiment analysis, user activity characterization, and event detection [53–55]. Consequently, tweet samples obtained from the Streaming API provide a valid basis for analyzing nighttime tweeting behavior. Fig 7 illustrates the temporal distribution of tweets by local hour in the study dataset. The hourly tweet volumes in this study align with the well-documented circadian pattern of Twitter activity in previous research [56]. This consistency supports the definition of nighttime as 10:00 p.m. to 6:00 a.m. local time, as these hours correspond to transition points in the circadian activity curve. Twitter was used as the sole data source in this study due to its large user base and the relatively structured nature of its content compared with other social media platforms. Future research could incorporate nighttime data from additional platforms, such as Facebook or Instagram, to provide a more comprehensive understanding of sleep pattern disruption. However, such integration would require additional efforts to harmonize differences in data sampling methods and data formats across platforms.

This study also has several limitations. First, tweets without geotags and those posted by non-individual users were excluded, which reduced the overall sample size and constrained the scope of the state-level analysis. As a result, some states did not have sufficient tweet samples to support robust analysis, leading to incomplete coverage across all U.S. states. In addition, the analysis was limited to nighttime tweet patterns at the state level, as uneven population distribution resulted in insufficient sample sizes for smaller geographic units. Future research could address these limitations by collecting larger samples over longer periods or by focusing on metropolitan areas with higher population densities to enable finer-scale analyses of sleeplessness issues. Second, for days with missing Twitter data, tweet counts and related metrics (e.g., nighttime tweet ratio) were interpolated using the average of the two adjacent days, which may introduce uncertainty into the results. Such missing temporal intervals are common in archived Twitter datasets. Future studies could mitigate this limitation by combining multiple Twitter data archives. Third, this study used the BERT-base-multilingual-uncased-sentiment model because of its broad language coverage, which allowed sentiment analysis to be applied to a larger set of tweets. However, the model was trained on approximately 629,000 pre-labeled product reviews [46], and differences in context between product reviews and nighttime tweets may affect classification accuracy. Future research could address this limitation by manually annotating a subset of tweets and training or validating multiple sentiment classification models, subject to available resources and labor constraints. Fourth, nighttime tweets provide an indirect, population-level social sensing signal related to potential sleeplessness, as operationally defined in this study, rather than a direct measure of individual-level sleep disturbance. Although nighttime posting suggests that a user was likely awake, it does not necessarily indicate clinically defined sleep disturbance, insomnia, poor sleep quality, or involuntary sleeplessness. Night-shift work, scheduled or automated posts, travel, and intentional late-night social media use may also contribute to nighttime activity. Our baseline-comparison approach helps reduce the influence of stable nighttime activity patterns by comparing pandemic-period nighttime tweet ratios with pre-pandemic baseline usage patterns for the same state and calendar month. Fifth, the baseline usage pattern was constructed using Twitter data from 2017–2018, whereas the pandemic period in this study covered March 2020 to June 2021. During this interval, social media usage patterns, user behaviors, geotagging practices, and platform policies may have changed independently of the pandemic. Therefore, some observed differences may partly reflect broader temporal trends in Twitter/X activity rather than pandemic-related sleep disturbance alone. The comparison with the baseline usage pattern should thus be interpreted as deviations from a historical pre-pandemic reference, rather than as a causal estimate of the pandemic’s independent effect. Last, the 10:00 p.m.–6:00 a.m. window was selected because the hourly tweet distribution showed a clear decline in posting activity beginning around 10:00 p.m. and remaining low through the early morning hours as shown in Fig 7. We acknowledge that tweets posted between 10:00 p.m. and 11:30 p.m. may include normal late-evening activity and should not be interpreted as direct evidence of insomnia or individual-level sleep disturbance. A stricter window, such as 12:00 a.m.–5:00 a.m., would reduce the nighttime tweet ratio to below 10% for most states. Given the minimum threshold of more than 30 geotagged tweets per state-day, this would correspond to fewer than three expected nighttime tweets per valid state-day, making daily nighttime tweet ratios more sensitive to one or two posts. Therefore, we retained the 10:00 p.m.–6:00 a.m. window to balance construct validity with state-level sample coverage. Accordingly, the nighttime tweet ratio should be interpreted as an indirect aggregate indicator related to potential sleeplessness or nighttime wakefulness, rather than as a direct measure of insomnia or clinical sleep disturbance.

## Conclusion

Using nighttime social media as a social sensing tool, this study examined population-level sleep disturbance patterns across U.S. states during the COVID-19 pandemic by analyzing the spatial, temporal, sentiment, and topical characteristics of nighttime tweets. Three distinct temporal patterns of sleep disturbance during the pandemic were identified, reflecting heterogeneous impacts across states: the “Single Peak” pattern, denoting early-stage disturbances; the “Multiple Peaks” pattern, indicating recurrent sleep disturbances; and the “Smooth” pattern, showing no clear disturbance. Correlations between nighttime tweet ratios and daily new COVID-19 case counts varied across states and pandemic stages. The findings further show that more negative emotional expression in nighttime tweets is associated with greater disruption of typical sleep patterns, as reflected by elevated nighttime tweeting activity. Compared with daytime tweets, nighttime tweets are more emotionally negative and are better indicators of people’s potential sleep disturbance. The pandemic was not the sole factor influencing sleep patterns during this period; other major events also contributed to sleep disturbance. Nighttime social media data provides a valuable means of identifying and tracking both short-term trending events, such as elections, social movements, and disasters, as well as the emergence of long-term events, exemplified by the COVID-19 pandemic. In addition, nighttime tweets were more likely to focus on local news and events, and greater diversity in topical attention across the population was associated with reduced negative emotional impact during the pandemic.

This study introduces and validates a novel social sensing approach that leverages nighttime social media big data to monitor and examine population-level patterns of potential sleep disturbance at large spatial and temporal scales. This framework offers a promising direction for future research on sleep-related behaviors, particularly in contexts where traditional data sources are limited or unavailable. Persistent late-night social media engagement in specific regions may signal disruptions to typical sleep patterns, with potential implications for daytime functioning at the population level. Accordingly, the nighttime tweet ratio can serve as a meaningful indicator for identifying locations experiencing sleep pattern disruption and associated declines in productivity. Beyond sleep-related research, this social sensing framework may be extended to examine other human activities and social dynamics during nighttime, including, but not limited to, crime patterns, disaster response, and social unrest.

## Supporting information

S1 Fig Monthly nighttime geotagged tweet counts in the United States from March 2020 to June 2021. (TIF)
